# Preparation of Fresh-Keeping Paper Using Clove Essential Oil through Pickering Emulsion and Maintaining the Quality of Postharvest Cherry Tomatoes

**DOI:** 10.3390/foods13091331

**Published:** 2024-04-26

**Authors:** Youwei Yu, Haochen Li, Yanfei Song, Biyu Mao, Shaoze Huang, Zhuoya Shao, Dingxian Wang, Kejing Yan, Shaoying Zhang

**Affiliations:** College of Food Science, Shanxi Normal University, Taiyuan 030031, China; 2030010210@sxnu.edu.cn (H.L.); 2130010218@sxnu.edu.cn (Y.S.); 2130010216@sxnu.edu.cn (B.M.); 2030010206@sxnu.edu.cn (S.H.); 2330010310@sxnu.edu.cn (Z.S.); 2330010308@sxnu.edu.cn (D.W.); 18835735152@163.com (K.Y.); sxnuzsy@163.com (S.Z.)

**Keywords:** fresh-keeping paper, clove essential oil, pickering emulsion, cherry tomatoes, freshness preservation

## Abstract

This study focused on developing a Pickering emulsion fresh-keeping paper that contained clove essential oil (CEO). Cherry tomatoes served as the test material for assessing the preservative efficacy of fresh-keeping paper. The results showed that Pickering emulsion had strong stability. Additionally, the fresh-keeping paper had a good antioxidant activity and sustained-release effect on CEO. In terms of the preservation effect, 0.75 wt% CEO Pickering emulsion paper reduced the decay incidence and weight loss of cherry tomatoes during 12-day storage. Fresh-keeping paper could also play a positive role in protecting the sensory index and color difference of tomatoes. It slowed the decline rate of soluble solid concentration (SSC) and titrable acid (TA). The vitamin C (Vc) and hardness of preserved tomatoes using fresh-keeping paper were maintained at a high level. The paper also inhibited the growth of microorganisms significantly. Therefore, 0.75 wt% CEO Pickering emulsion fresh-keeping paper displayed considerable potential for application in the preservation of postharvest fruits and vegetables. It is a novel fruit and vegetable preservation material worthy of development.

## 1. Introduction

Cherry tomatoes (*Solanum lycopersicum var. cerasiforme*), an annual herb, belong to the tomato genus of the Solanaceae family. Their fruits are rich in nutrients such as carotenoids, ascorbic acid, total phenols and flavonoid [1], having the functions of moisturizing, quenching thirst, strengthening the stomach and increasing appetite. However, as a climacteric fruit, harvested cherry tomatoes will undergo sensory quality deterioration and decay during storage [2], leading to a decrease in nutritional value and commodity value. Therefore, it is really important to preserve cherry tomatoes fresh after harvest. Traditional methods such as chemical preservatives may maintain fruit and vegetable freshness. Although chemical preservatives can achieve the effect of preservation, they may produce pesticide residues [3,4], affecting the health of consumers. The use of plastic cling film preservation is also a common method of preservation. Plastic cling film has the advantages of high strength and convenient use. However, degrading plastic products is usually difficult and there is the problem of environmental pollution. Hence, it is necessary to develop novel methods and materials to extend the shelf life of postharvest fruits such as cherry tomatoes and reduce the risks of food safety [5]. 

As the policies of “plastic restriction” and “carbon neutrality” are implemented, the development of biodegradable food packaging materials with high performance, low pollution, and suitability for industrial production has become a current trend [6]. Preservative paper is a novel fresh-keeping material obtained through coating the preservative to paper. It has the characteristics of high performance, low pollution, and degradability. Meanwhile, it also has the characteristics of being light weight, small in size, high folding resistance, high flexibility, strong barrier and high permeability. Fresh-keeping paper with preservatives can be used as a novel material to preserve fruits and vegetables [7]. In this study, clove essential oil (CEO) was used as a preservative in fresh-keeping paper. CEO is a common preservative with broad-spectrum antibacterial activity, widely used in food, medicine, cosmetics and other fields [8]. It contains a large number of bioactive compounds, mainly including eugenol and its derivatives, styrene-acrylic compounds, and a small amount of β-caryophyllene and α-humulene organic compounds [9]. They have antibacterial and antifungal properties [10]. So, CEO is considered to be a natural preservative and antioxidant [11]. Though CEO has good antioxidant and antibacterial effects, it shows short preservation time and poor effect owing to its volatility if it is directly coated on fresh-keeping papers [12].

Pickering emulsions are surfactant-free emulsions, inhibiting droplet aggregation and having high stability. Their stability is achieved through the adsorption of solid particles on the two-phase interface to form a mechanical barrier and change the steric hindrance between particles, which is a thermodynamically irreversible process. Pickering emulsions currently have a wide range of applications in the food field, including preparing smart food films, delivering bioactive substances and preventing lipid oxidation. As for delivering bioactive substances, Pickering emulations can form a solid layer around CEO via colloidal particles to block the oil/water phase, so the emulsions were stabilized and the release of the essential oil was limited [13]. Compared with traditional surfactant emulsions, Pickering emulsions show higher stability and higher resistance to essential oil volatility [14]. Hence, Pickering emulsions can be used to encapsulate essential oil, so a sustained-release of essential oil was achieved [15]. In this study, Pickering emulsion was prepared with zein and pectin. Zein is a natural macromolecular protein derived from corn endosperm. Its structure contains 75% lipophilic amino acid residues and 25% hydrophilic amino acid residues. Most of the amino acid residues in zein are hydrophobic. Therefore, this property gives zein the advantage of delivering hydrophobic bioactivity [16]. Zein can be self-assembled to form nanoparticles by antisolvent precipitation, which can be used to prepare hydrophobic delivery carriers such as essential oil [17]. Nevertheless, there are still some problems in the stability and application of zein. Consequently, pectin solves the shortcomings of its lack of stability and limited application conditions.

So far, research on the essential oil encapsulated with Pickering emulsions has mostly focused on preparation and characterization [16], and there are few reports on functional fresh-keeping paper prepared by embedding essential oil in Pickering emulsions. In our research, CEO encapsulated in Pickering emulsions was coated on bibulous papers as a preservative to produce fresh-keeping papers with antioxidant, bacteriostatic, and preservative effects. The prepared papers could achieve the sustained release of CEO, thus extending the shelf life of cherry tomatoes. This research might provide a reference for the use of environmentally friendly storage methods to preserve fruits and vegetables.

## 2. Materials and Methods

### 2.1. Materials

Cherry tomatoes (*Lycopersicon esculentum* Mill. cv. Qianxi) were purchased from Wangda Agricultural Products Market (Taiyuan, China). CEO was provided by Ji‘an Linyuan Fragrance Co., LTD. (Ji‘an, China). Zein was purchased from Nanjing Dulai Biotechnology Co., LTD. (Nanjing, China). Citrus pectin was purchased from Yuzhong BioEngineering Co., LTD. (Zhengzhou, China). Fresh-keeping bags were purchased from the Jinfen Plastic Industry (Cangzhou, China). The reagent, 1, 1-Diphenyl-2-picrylhydrazyl (DPPH) was purchased from Shanghai Yuanye Biotechnology Co., LTD. (Shanghai, China). All other chemical reagents were of analytical grade.

### 2.2. Preparation of CEO Pickering Emulsion Fresh-Keeping Paper

0.45 wt% zein was dispersed in 80% ethanol solution and the resultant solution was sheared at 10,000 r/min within 1 min using a homogenizer (FSH-2B, Changzhou Fang Ke Instrument Co., LTD., Changzhou, China). Pectin (0.3 wt%) was dissolved in deionized water at 70 °C [18]. Zein nanoparticles were dispersed in the pectin solution while stirring at 800 r/min. Afterward, the solution was stirred to evaporate ethanol, and the pH of the composite colloidal particle dispersions was adjusted by HCl solution (0.1 M) to 4.0. The deionized water with pH 4 was added to ensure the original mass. CEO was added into the composite colloidal particle dispersions and clipped at a rate of 14,000 r/min for 4 min. Then Pickering emulsion was prepared. The emulsion was stored at room temperature (25 °C).

The bibulous papers were soaked in the prepared CEO Pickering emulsion and removed after 5 min. A piece of bibulous paper was placed at the bottom of the fresh-keeping paper to stick them together. The CEO Pickering emulsion fresh-keeping papers were dried at room temperature (25 °C).

### 2.3. Sample Treatment

Cherry tomatoes with the same color, maturity, hardness and without mechanical damage and pests, were selected for experimental research. A piece of fresh-keeping paper was put on the top of cherry tomatoes and a fresh-keeping plastic bag was used to entirely wrap the cherry tomatoes and paper. The closure method of the bag was folding. Then the fresh-keeping plastic bag that is regularly used to pack fruits and vegetables (63.2 cm × 53.7 cm, thickness is 0.013 mm, material is high-density polyethylene), the fresh-keeping paper, and cherry tomatoes were put into a 38 × 30 × 11 cm plastic basket. Finally, all samples were stored at room temperature (25 ± 1 °C) and 95 ± 2% RH.

### 2.4. Screening the Best CEO Concentration

Four different weight percentages of CEO Pickering emulsions were prepared to study the effect of CEO concentrations on the preservation of cherry tomatoes. Quantities of 0, 0.50, 0.75 and 1.00 wt% of CEO were added to the composite colloidal particle dispersions to prepare the Pickering emulsions. The bibulous papers were soaked in the prepared emulsions to obtain CEO fresh-keeping papers with different weight percentages. The selected 50 cherry tomatoes were placed under the prepared fresh-keeping papers for 15 days and decay incidence was measured. The experiment was repeated three times. According to the preliminary screening, the fresh-keeping paper with the concentration of CEO 0.75 wt% had the lowest decay incidence and showed the best preserving effect.

### 2.5. Characterization of Pickering Emulsion and Fresh-Keeping Paper

The Pickering emulsions and fresh-keeping papers were characterized using a series of indicators to study the stability and sustained-release properties (stability of Pickering emulsions, stability of Pickering emulsions, ζ-potential & particle size analyzes, optical microscopy observation, scanning electron microscopy observation, dynamic process of CEO sustained-release and antioxidant assay).

#### 2.5.1. Interfacial Tension of Emulsions

Interfacial tension of the different PG: Chi emulsions was measured using a Krüss BPT Mobile tensiometer (BPT Mobile, Krüs, Germany) according to the standard Wilhelmy’s plate method (Pareta & Edirisinghe, 2006) [19]. All measurements were performed in triplicate at 22 ± 1 °C for 45 min until a constant value was achieved [20].

#### 2.5.2. Stability of Pickering Emulsions

The prepared composite colloidal particle dispersion that had 0 wt% CEO and 0.75 wt% CEO Pickering emulsion was placed in glass bottles. The coagulation and clarity were observed from day 0 to day 4.

Centrifuging samples assessed the centrifugal stability of samples for 15 min at 8000 rpm [21]. The creaming index (CI) was determined as follows:

CI = V_s_/V_0_ × 100

where V_s_ was the volume of supernatant after centrifugation, and V_0_ was the initial volume of Pickering emulsion.

#### 2.5.3. ζ-Potential & Particle Size Analyzes

The assessment of droplet size distributions and zeta potentials for the diverse emulsions were conducted using dynamic light scattering via a Zetasizer Nano ZS90 instrument (ZS90; Malvern Instruments Ltd, Worcestershire, UK). Deionized water was employed to dilute the sample to achieve a concentration of 0.1% *w*/*v*. The droplet size was expressed by the z-average diameter resulting from the mean of 3 sequential measurements. These measurements were executed at room temperature of 25 °C, and each experiment was iterated thrice [22].

#### 2.5.4. Optical Microscopy Observation

The sample (40 μL) was dropped onto a clean glass slide and observed using an optical microscope (CX31RTSF, Shanghai Dending International Trade Co., LTD, Shanghai, China) under magnifications of 100× and 200× [23].

#### 2.5.5. Scanning Electron Microscopy (SEM) Observation

Surface morphologies of papers that contained plain papers, papers soaked in composite colloidal particle dispersion, and papers soaked in 0.75 wt% CEO Pickering emulsion, were observed using a scanning electron microscope (Gemini SIGMA 300, Oberkochen, Germany). Prior to observation, the papers were sputter coated with gold [23].

#### 2.5.6. Dynamic Process of CEO Sustained-Release 

A total of 0.5 mg of CEO was transferred to a 100 mL volumetric bottle using anhydrous ethanol. CEO standard solutions 40, 50, 60, 70 and 80 μL were measured and then fixed to 10 mL with anhydrous ethanol to obtain 0.020, 0.025, 0.030, 0.035 and 0.040 μg/mL standard solutions. With anhydrous ethanol as a blank control, the concentration of CEO was measured at 280 nm (maximum absorption wavelength). The standard curve is y = 0.0051x + 0.0651 (R^2^ = 0.9968).

A weight of 0.75 wt% CEO was dissolved in anhydrous ethanol of the same quality as CEO Pickering emulsion. Next, ordinary bibulous papers were soaked in the solution for 5 min. Then, 0.75 wt% CEO Pickering emulsion was prepared, and bibulous papers were dipped in the emulsion for 5 min as well.

The anhydrous ethanol group and 0.75 wt% CEO group were placed in the air. After 4, 24, 48, 72 and 96 h, the papers of the two groups were respectively cut into 2 × 2 cm^2^ sizes. The little papers were put into 10 mL of 95% ethanol [14]. The absorbance of the supernatant was measured at 280 nm using a double-beam UV-visible spectrophotometer (TU-1901, Beijing Puxi General Instrument Co, Beijing, China) [24], and the measured absorbance values were recorded.

#### 2.5.7. Antioxidant Assay

The antioxidant activity of fresh-keeping paper was measured by a scavenging assay against the DPPH radical. The paper was cut into small pieces of 20 mm × 30 mm and placed in 10 mL deionized water. The sample was shaken on a shaker for 12 h, and then it was centrifuged at 5000× *g* for 10 min. The supernatant of 2.0 mL mixed with DPPH ethanol solution of 0.17 mM, and incubated in the dark at room temperature for 30 min [25]. The absorbance of the reaction solution at 517 nm was determined.

DPPH scavenging activity = 1 − A_1_/A_0_ × 100%

where A_0_ and A_1_ are the absorbances of the blank group and the sample group, respectively.

### 2.6. Preservation Effect of Fresh-Keeping Paper on Cherry Tomatoes

To further study the effect of fresh-keeping paper on cherry tomatoes, 0 wt% (control group) and 0.75 wt% CEO fresh-keeping papers (0.75 wt% CEO group) were used to evaluate the quality of cherry tomatoes. During the storage period of cherry tomatoes, the quality evaluation was respectively conducted at 0, 3, 6, 9 and 12 days. This included measurement of physical and chemical properties (incidence of decay, sensory evaluation, weight loss, soluble solid concentration, titrable acids, vitamin C, and hardness) and microbiological analysis (total number of aerobic bacteria, molds and yeasts).

#### 2.6.1. Incidence of Decay

The total number of cherry tomatoes and decay were counted, and the incidence of decay was determined using the following equation:

incidence of decay = a / b × 100%

where a denotes the number of decayed cherry tomatoes, b denotes the total number of cherry tomatoes.

#### 2.6.2. Sensory Evaluation

Ten expert group members (5 males and 5 females, aged from 20 to 50) with sensory experiment and quantitative description analysis experience were recruited from our laboratory. They were trained for 4 weeks (20 min per day) to describe the taste characteristics of cherry tomato samples, including aroma and taste as evaluation indices, and distinguish their differences. Aroma and taste were used as the evaluation indicators. Each indicator had a full score of 50 points for a total of 100 points. Sensory evaluation was performed in a sensory panel room at 22 ± 2 °C with 40–80% humidity. All the panelists rinsed their mouths with boiled water, and the next sample was tasted after a rest for 15 s. The results evaluated by a single panelist differed by <20% [26].

#### 2.6.3. Color Difference

To study the influence of CEO Pickering emulsion fresh-keeping paper on the cherry tomatoes, a random evaluation of the color of the cherry tomatoes was conducted employing the colorimeter (NH310, ThreeNH Technology, Shenzhen, China). A total of 12 measurements were carried out for each group. L* is defined as luminosity, a* (±red-green) signifies the redness, and b* (±yellow-blue) conveys the yellowness [27]. The overall color variation (ΔE) is computed using the following equation:

$$\Delta E=\sqrt{{\left(\Delta L*\right)}^{2}+{\left(\Delta a*\right)}^{2}+{\left(\Delta b*\right)}^{2}}$$

where ΔL, Δa, and Δb are the color parameter differences between samples and standard samples in each group.

#### 2.6.4. Weight Loss

During storage, the weight loss of cherry tomatoes was determined by analyzing the mass of all cherry tomatoes at the beginning and every 3 days. Weight loss was expressed as a percentage (%). The rate of weight loss was determined using the following equation:

Weight loss = (m_0_ − m_t_) / m_0_ × 100%

where m_0_ denotes the initial weight of the sample, m_t_ denotes the weight of the sample on days 3, 6, 9 and 12.

#### 2.6.5. Soluble Solid Concentration (SSC)

A hand-held digital refractometer (WYT-1, Qingyang Optical Instrument Co., Ltd., Chengdu, China) was used to measure the content of soluble solids in preserved samples. Each group of samples was sliced and squeezed to release the juice (>2 drops) and placed in the refractometer. The result was expressed as a percentage (%) [28].

#### 2.6.6. Titratable Acid (TA)

Cherry tomatoes (25 g) were ground and strained with a clean cloth. The filtrate was poured into a beaker and the appropriate amount of distilled water was added. The diluted filtrate was filtered again and poured into a 100 mL volumetric bottle with constant volume. Then, 25 mL of filtrate was poured into a conical bottle. Two drops of phenolphthalein indicator were added. Next, the filtrate was titrated with 0.1 mol/L NaOH solution until the color turned pink [29]. The titratable acid content was determined using the following equation:

X = (v_1_ × 0.1 × 0.067 × v_2_ × 100) / (m × v_3_)

where X denotes the content of titratable acid, v_1_ denotes the consumption of NaOH, v_2_ denotes the total volume of the filtrate, m denotes the initial weight of the sample, and v_3_ denotes the amount of filtrate required during the experiment.

#### 2.6.7. Vitamin C (Vc)

The content of Vitamin C was determined by 2, 6-dichlorophenol titration according to the National Standard of China (GB 5009.86–2016 [30]) issued by the National Health and Family Planning Commission of the People’s Republic of China. The Vc content was determined using the following equation:

X = ((v_1_ − v_2_) × k × v_3_ × 100) / (m × v_4_)

where X denotes the content of Vc, v_1_ denotes the consumption of 2, 6-dichlorophenol, v_2_ denotes the volume of 2, 6-dichlorophenol consumed by the titration blank group, k denotes the volume of Vc oxidized by 1 mL 2, 6-dichlorophenol, v_3_ denotes the total volume of the extracting solution, m denotes the initial weight of the sample, and v_4_ denotes the volume of filtrate. 

#### 2.6.8. Hardness

The hardness of cherry tomatoes was measured using a texture analyzer (TA. XT. plus SMS, Godalming, UK) equipped with a cylindrical p/2 probe. Each sample was placed on the platform and was penetrated by the probe for 2 mm at a speed of 1.00 mm/s. Each measurement was replicated four times for the same sample [28].

#### 2.6.9. Microbiological Analysis

Microbiological analysis is an important basis for judging the spoilage degree of fruits and vegetables. The total number of colonies in cherry tomatoes was determined according to the National Standard of China (GB4789.2-2022 [31]).

A total pf 5 cherry tomatoes were randomly selected and crushed into a paste. Then, 5 g of obtained paste was placed in a conical flask containing 45 mL of sterile saline and shaken thoroughly for 3 min, followed by decimal gradient dilution of the suspension [2]. A series of decimal dilutions were prepared and spread over plate count agar (PCA) and chloramphenicol yeast glucose agar media (YGC) for mesophilic aerobic bacteria and yeast and molds, respectively. Plates for mesophilic aerobic bacteria were incubated at 30 °C for 3 days, and yeast and molds at 20 °C for 5 days. Results were expressed as log of colony-forming units per gram of fresh weight (log CFU/g). Three replicates were completed for each treatment and storage time [32]. 

### 2.7. Statistical Analysis

Data analysis was conducted using SPSS Statistics 27 software, and the results were presented as mean ± standard deviation. Subsequently, the data were subjected to a one-way analysis of variance (ANOVA) followed by post-hoc analysis using Duncan’s multiple range test. Statistical significance was considered at a threshold of *p* < 0.05.

## 3. Results and Discussion

### 3.1. Characterization of Pickering Emulsion and Fresh-Keeping Paper

#### 3.1.1. Interfacial Tension of Emulsions

Interfacial tensions of the different samples are presented in Table 1. The interfacial tension of Pickering emulsion group with CEO (0.75 wt%) is lower than that of composite colloidal particle solution (0 wt%). The presence of oil released in the system could lead to a decrease in the interfacial tension of emulsions, since the interfacial tension of the oil is lower than those showed by the combination of zein and pectin [20].

#### 3.1.2. Stability of Pickering Emulsions

**Figure 1:**
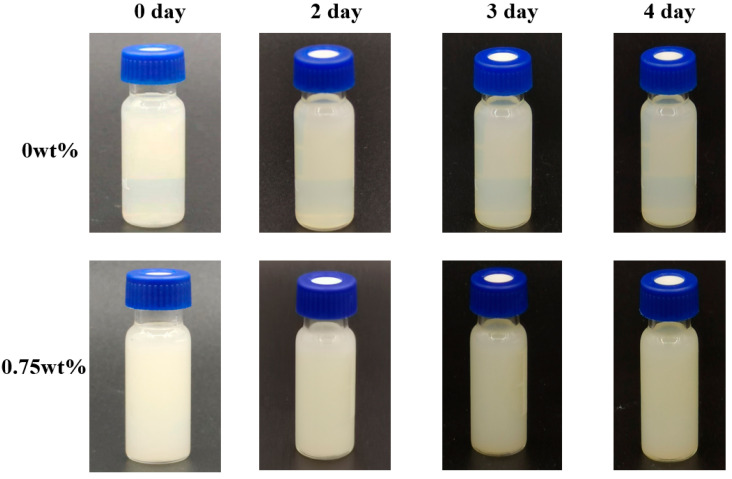
Appearance of composite colloidal particle dispersion (0 wt%) and Pickering emulsion (0.75 wt%).

As is shown in Figure 1, from day 0 to day 4, the appearance of composite colloidal particle dispersion and Pickering emulsion remained stable and uniform without any demulsification or delamination. The appearance of the Pickering emulsion group with CEO (0.75 wt%) was milky, and the composite colloidal particle dispersion without CEO (0 wt%) was clearer and more transparent. The reasons might be as follows. After the addition of CEO, the interfacial tension of oil-water interface in the emulsion was reduced, so that the oil phase was dispersed into the water phase to form a milky white stable liquid [20].

The centrifugal stability of the different samples is presented in Table 1. The CI of 0.75 wt% group was 98.85 ± 0.165%, and that of the control group was 96.88 ± 0.462%. They all had strong stability, but 0.75 wt% emulsion had stronger stability.

#### 3.1.3. ζ-Potential & Particle Size Analyzes

ζ potential represents the likelihood of droplet aggregation. The larger absolute value of ζ-potential suggests a more stable system [14]. According to Table 1, the potential values of 0.75 wt% group was higher, so the system had strong stability. The reason was that 0.75 wt% group contained negatively charged pectin. ζ potential was positively correlated with pectin due to its negative charge [33].

As depicted in Table 1, the average particle size of the 0.75 wt% group was 463.47 ± 4.177, and particle size of the control group was 399.63 ± 3.571. The larger droplet size of 0.75 wt% might be due to the increased surface coverage of large particles in Pickering emulsion.

#### 3.1.4. Optical Microscopy Observation

**Figure 2:**
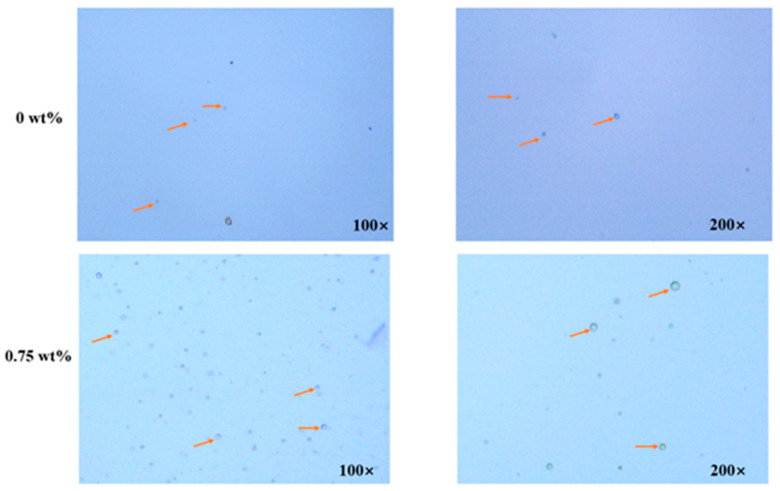
Optical microscope images of composite colloidal particle dispersion (0 wt%) and CEO Pickering Emulsion (0.75 wt%).

Optical microscopy showed that the droplets of the two samples were uniformly spherical (Figure 2). The sample drops had good dispersibility, clear boundaries, and no condensation phenomena. This might be due to the fact that composite colloidal particle dispersion and Pickering emulsion had a high ζ potential value, which provided a strong electrostatic repulsion force and enhanced the resistance to droplet aggregation [34]. Meanwhile, high methoxyl pectin (HMP) is used to make composite colloidal particle dispersion and Pickering emulsion. It is an amphiphilic substance, containing hydrophilic hydroxyl groups and methoxy groups. The pectin in samples can be adsorbed on the oil-water interface to reduce the interfacial tension and thus form stable droplets. The added HMP can also increase the viscosity of samples to reduce the rate of coagulation [18]. Notably, the droplets of Pickering emulsion added with 0.75 wt% CEO had denser oil droplet accumulation and tended to form a three-dimensional gel-like droplet network structure, which might have more stable structural characteristics [16].

#### 3.1.5. Scanning Electron Microscopy (SEM) Observation

**Figure 3:**
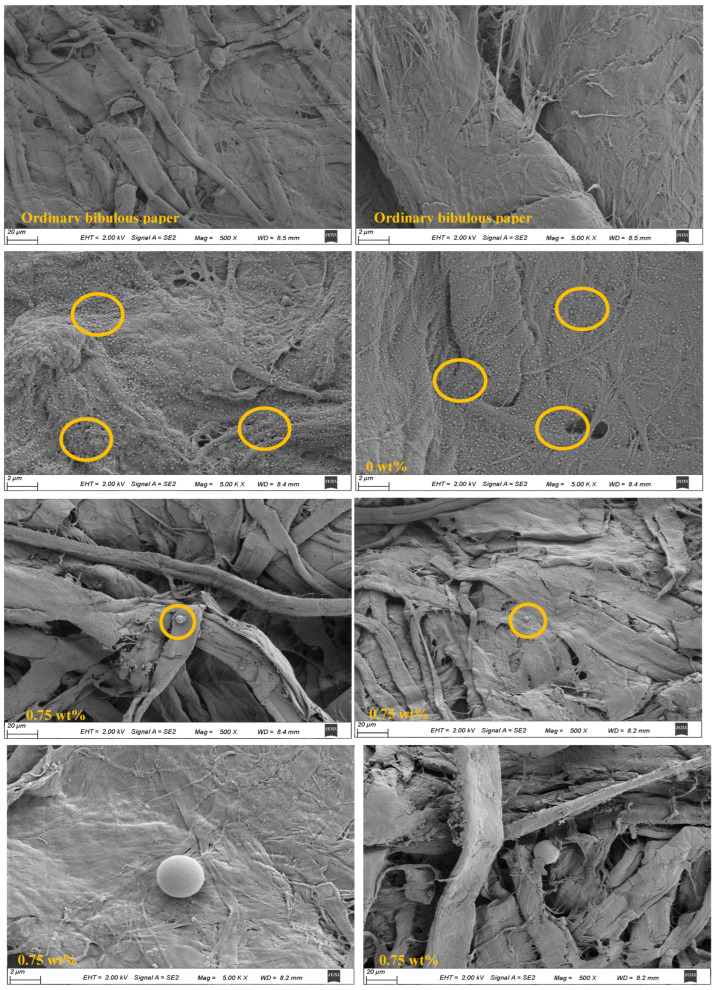
SEM images of ordinary bibulous paper, bibulous paper soaked in composite colloidal particle dispersion (0 wt%), and Pickering emulsion fresh-keeping paper (0.75 wt%). The yellow circles’ role is to highlight the density and distribution of particles.

SEM photos indicated that there were no particles on the ordinary absorbent paper, but particles can be observed in bibulous paper soaked in composite colloidal particle dispersion (0 wt%) and 0.75 wt% CEO group (Figure 3). Both particles were uniformly spherical, and the spherical droplets were emulsion droplets. The droplets of the bibulous paper group soaked in the composite colloidal particle dispersion were densely distributed, and there was a phenomenon of mutual condensation. The droplets in 0.75 wt% CEO group were evenly distributed on the paper without aggregation. The reason for this might be that the hydroxyl and carboxyl groups of CEO enhance the interaction among droplets, thus improving the stability of droplets. Accordingly, the droplets in the emulsion are evenly distributed [35].

#### 3.1.6. Dynamic Process of CEO Sustained-Release

**Figure 4:**
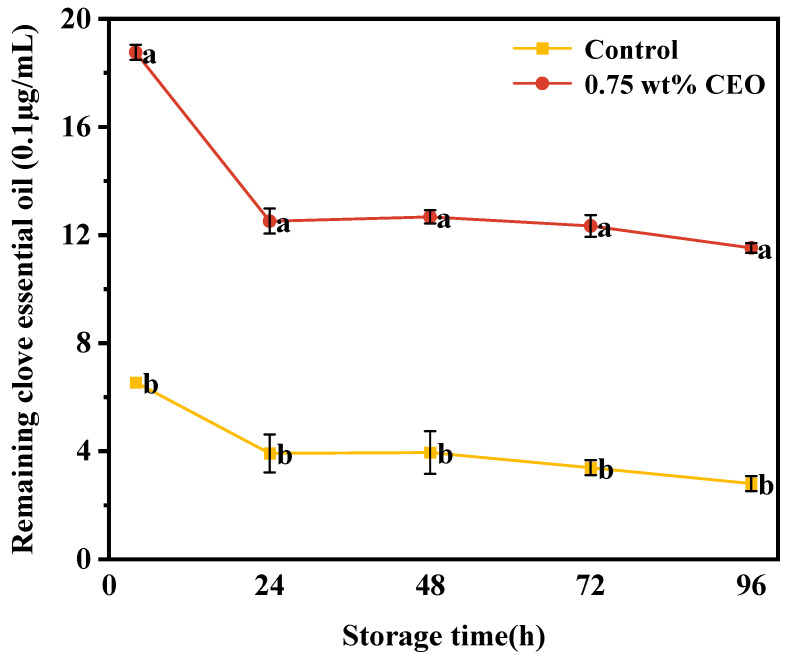
During a 96 h storage period at 25 °C, retention of essential oil in anhydrous ethanol group (Control) and CEO Pickering emulsion fresh-keeping paper (0.75 wt%). Different superscript letters (a, b) represent statistically significant differences (*p* < 0.05).

As shown in Figure 4, after sustained release for 4, 24, 48, 72 and 96 h, the amount of loaded essential oil in the anhydrous ethanol group and Pickering emulsion paper was detected. The retention of essential oil in anhydrous ethanol group and 0.75 wt% CEO group showed a downward trend, which was related to the strong volatility of CEO itself. From 4 h to 24 h, the retention of essential oil in papers was greatly reduced, and explosive release occurred. This is mainly due to a large amount of CEO absorbed on the surface of papers [14]. However, the release rate of essential oils decreased in the middle and late periods, and then the amount of essential oils on papers became reduced. In comparison to the anhydrous ethanol group, the retention of essential oil in 0.75 wt% CEO group was larger, and the release rate of essential oil was slower as well. This might be due to the zein and pectin fresh-keeping paper forming a stable protective layer that could protect the CEO and achieve the sustained-release process. This result is similar to Zhang Wei et al. [16] who applied zein and pectin to achieve lycopene protection. 

#### 3.1.7. Antioxidant Assay

CEO has strong antioxidant properties, so the fresh-keeping paper that can achieve slow release of essential oil also has strong antioxidant properties. Table 1 illustrates DPPH scavenging activity of 0 wt% group and 0.75 wt% group. DPPH scavenging activity of 0 wt% was 55.2 ± 0.019%, and the capacity of 0.75 wt% group to scavenge the free radicals was significantly increased, reaching 87.7 ± 0.042%. The large number of unsaturated double bonds in eugenol, the main bioactive compound in CEO, has the ability to scavenge free radicals. Moreover, the hydroxyl groups of eugenol are usually bound to the carbon atom of the aromatic ring, which facilitates the provision of hydrogen atoms as radical acceptors that interact with free radicals to form stable products and prevent oxidative damage [36]. Therefore, the CEO contained in the fresh-keeping paper had a strong antioxidant capacity.

### 3.2. Preservation Effect of Fresh-Keeping Paper on Cherry Tomatoes

#### 3.2.1. Incidence of Decay

**Figure 5:**
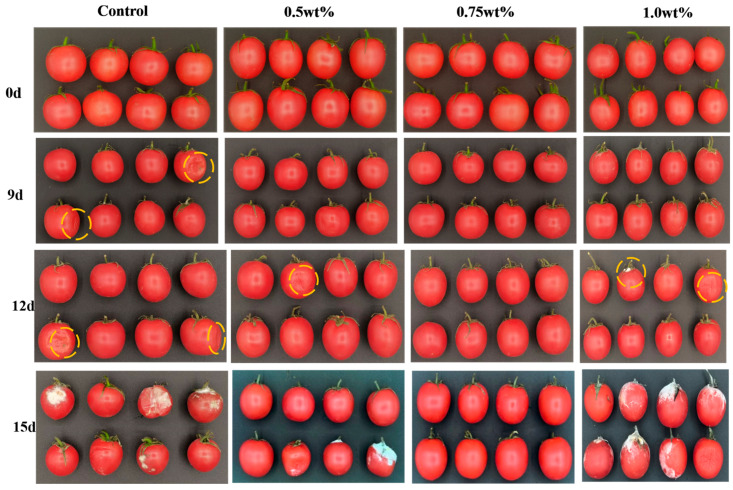
Photographs of cherry tomatoes preserved in Pickering emulsion papers with different concentrations of CEO during a 15-day storage period at 25 °C. The yellow circles’ role is to highlight the rot on the cherry tomatoes.

Cherry tomatoes were preserved using 0, 0.5, 0.75 and 1.0 wt% CEO Pickering emulsion fresh-keeping papers at room temperature for 15 consecutive days. The decay degree of cherry tomatoes in each group was observed at 0, 9, 12 and 15 days, respectively. The experimental results are shown in Figure 5. With the extension of storage time, the number of rotten fruits increased. On day 0 of storage, there were no rotten fruits in each group. However, some fruits showed rot on the 9th day. The most serious rotting was in the control group (0 wt%). After 15 days of continuous storage, the 1.0 wt% concentration of fresh-keeping paper group had the most serious decay. Control group and 0.5 wt% CEO group had similar levels of decay, while the 0.75 wt% CEO group was able to inhibit the decay incidence.

**Figure 6:**
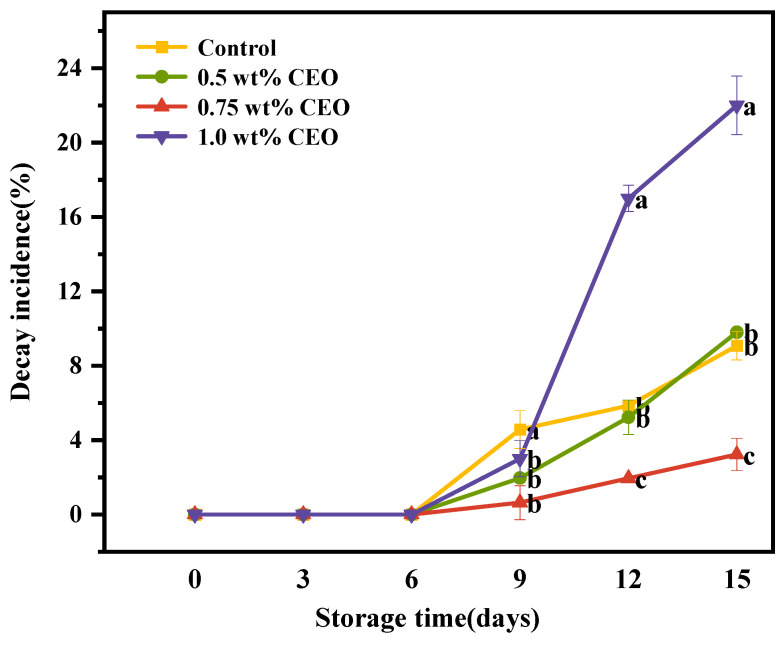
During a 15-day storage period at 25 °C, the effect of different concentrations of CEO Pickering emulsion fresh-keeping papers on the incidence of cherry tomatoes decay. Different superscript letters (a–c) represent statistically significant differences (*p* < 0.05).

As depicted in Figure 6, with the extension of the storage time of cherry tomatoes, the decay incidence of each group showed an increasing trend. Among them, the highest decay incidence of 23.08% was observed in the sample with 1.0 wt% CEO after 15 days of storage. Meyer et al. demonstrated that tomatoes were sensitive to CEO. CEO, especially its primary constituent eugenol, has been reported to have phytotoxic effects [37]. The decay incidences of control group and 0.5 wt% were similar. Their decay incidences reached 9.62% and 9.80%, respectively, after 15 days of storage. The 0.5 wt% group did not achieve the expected effect, which was due to the concentration of CEO in the 0.5 wt% group being lower. The decay incidence of 0.75 wt% was only 2%, and the inhibition effect was the best. It showed that in the proper concentration range, the CEO can inhibit cherry tomato rotting. Suhua Yang et al. [38] found that within a certain range, CEO can inhibit the rot of strawberries compared with control group, which was consistent with the results of this study. In this study, the paper prepared with Pickering emulsion of CEO could achieve sustained release of essential oil. During the 15-day storage process, the CEO achieved sustained release, thus extending the freshness of postharvest cherry tomatoes.

#### 3.2.2. Sensory Evaluation

**Figure 7:**
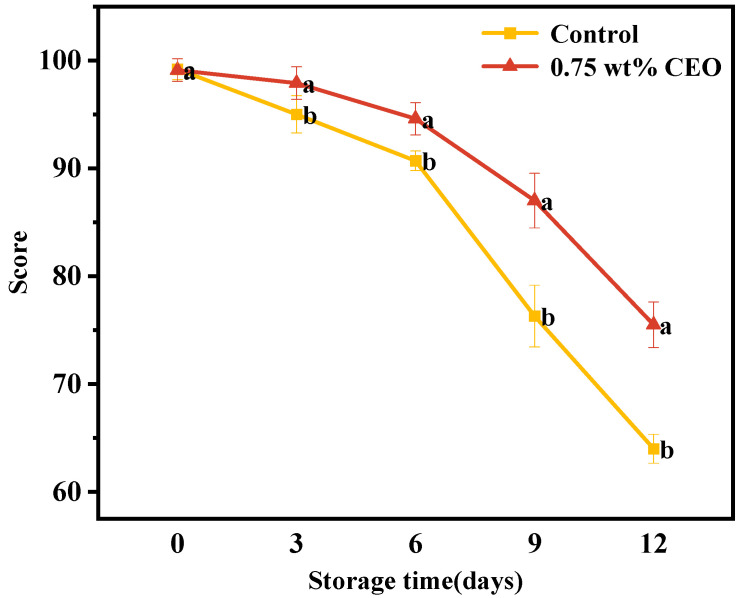
During a 12-day storage period at 25 °C, the effect of CEO Pickering emulsion fresh-keeping paper on sensory evaluation scores of cherry tomatoes. Different superscript letters (a, b) represent statistically significant differences (*p* < 0.05).

As shown in Figure 7, the sensory scores of cherry tomatoes showed a downward trend during storage process. However, the score of 0.75 wt% group dropped from 99.1 to 75.5 points, and the control group dropped to 64 points. The scores of 0.75 wt% group were significantly higher than control group (*p* < 0.05). The results showed that the quality of cherry tomatoes continued to decline during storage, but 0.75 wt% group was more conducive to the preservation of cherry tomatoes [26]. And the tomatoes were in essential oil vapor which cannot negatively affect the taste and aroma of the fruits.

#### 3.2.3. Color Difference

**Figure 8:**
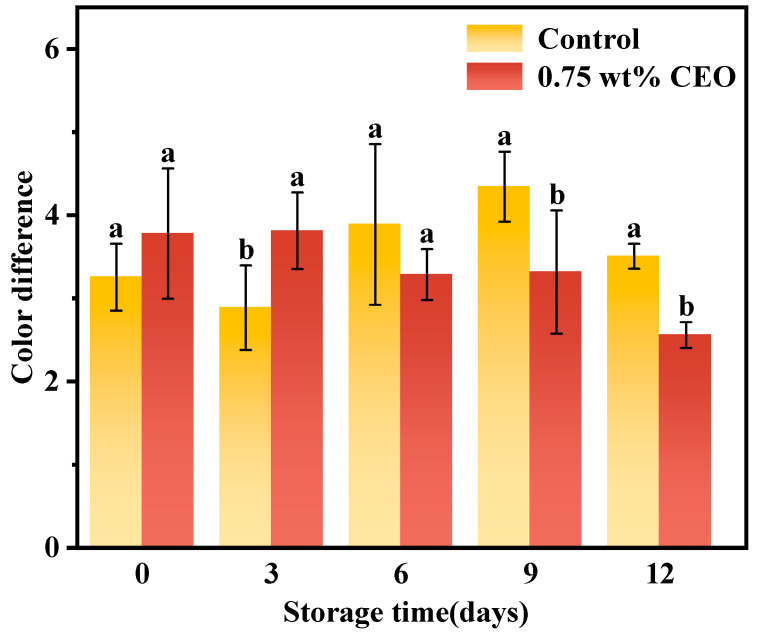
During a 12-day storage period at 25 °C, the effect of CEO Pickering emulsion fresh-keeping paper on color difference of cherry tomatoes. Different superscript letters (a, b) represent statistically significant differences (*p* < 0.05).

According to Figure 8, the effects of 0.75 wt% CEO group on color differences were obviously better than those of control group (*p* < 0.05) in the later stages of storage. Results on the 12th day showed that the color difference of 0.75 wt% CEO group was only 73% of that of control group. The skin color of the cherry tomato is closely related to the oxidative degree of internal nutrient substances, such as carotenoids and xanthophylls [27]. Because the fresh-keeping paper had a strong antioxidant effect, the content of nutrients was relatively stable and ΔE was lower.

#### 3.2.4. Weight Loss

**Figure 9:**
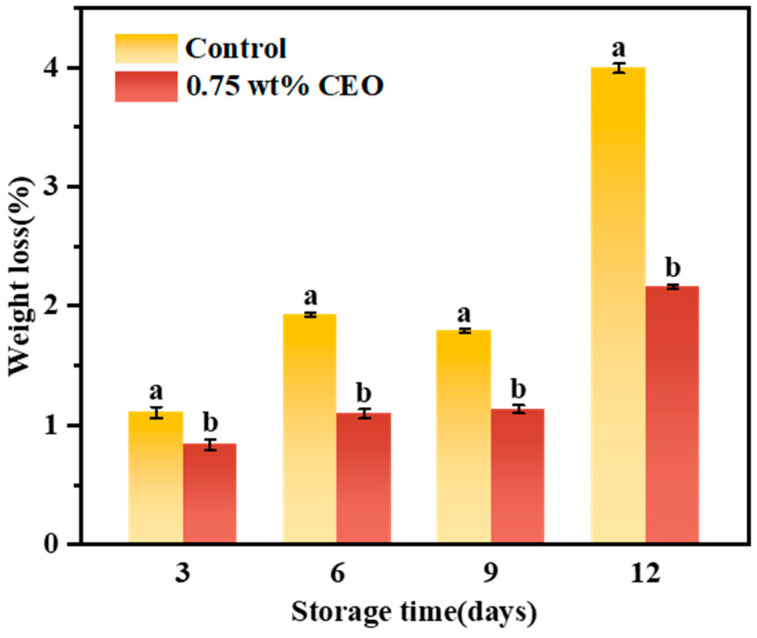
During a 12-day storage period at 25 °C, the effect of CEO Pickering emulsion fresh-keeping paper on weight loss of cherry tomatoes. Different superscript letters (a, b) represent statistically significant differences (*p* < 0.05).

Figure 9 illustrates the weight loss rate of cherry tomatoes. With time, the weight loss of control group and 0.75 wt% CEO group showed an increasing trend. On day 3, there was a significant difference in weight loss between control group (1.11%) and 0.75 wt% CEO group (0.84%) (*p* < 0.05). On day 6, the weight loss of control group reached 1.93%, while that of the 0.75 wt% CEO group was only 1.10%. On the 12th day of storage, the weight loss rate of control group increased sharply, it reached 3.99%. Most cherry tomatoes had lost their commercial value. The weight loss rate of 0.75 wt% CEO group was 2.17%. Therefore, the weight loss rate of control group was higher, and 0.75 wt% CEO Pickering emulsion fresh-keeping paper effectively inhibited the increase of the weight loss rate of cherry tomatoes.

Since cherry tomatoes will still carry out physiological activities such as respiration and metabolism after picking, control group and 0.75 wt% CEO group show the phenomenon of weight loss after storage for a while. Nonetheless, the weight loss rate of control group was higher, indicating that CEO volatilized on Pickering emulsion paper could effectively slow down the water transpiration of cherry tomatoes. The result was similar to Luesuwan et al. who study the preservation of grapes by CEO [39]. Hence, the water evaporation rate of cherry tomatoes in 0.75 wt% CEO group was greatly reduced. Accordingly, the weight loss rate of 0.75 wt% CEO group slowly increased.

#### 3.2.5. Soluble Solid Concentration (SSC)

**Figure 10:**
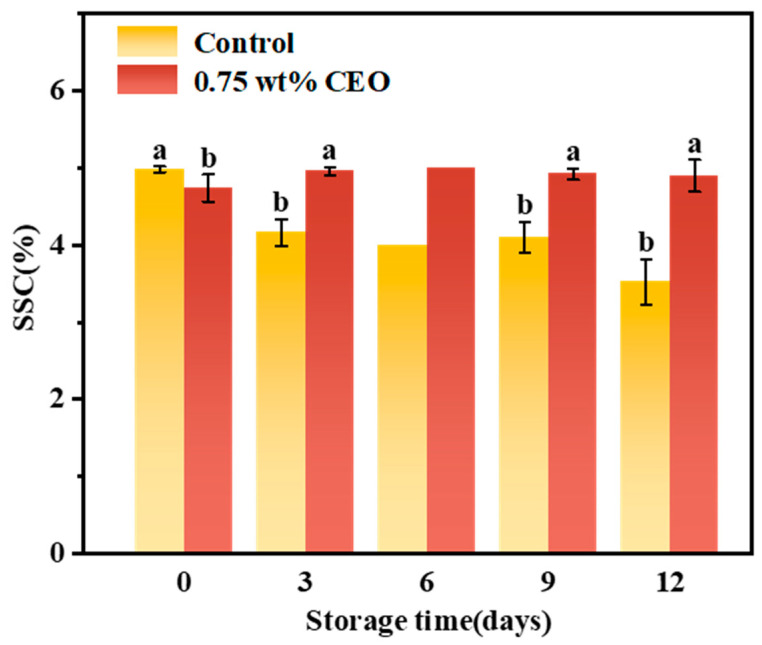
During a 12-day storage period at 25 °C, the effect of CEO Pickering emulsion fresh-keeping paper on soluble solid concentration (SSC) of cherry tomatoes. Different superscript letters (a, b) represent statistically significant differences (*p* < 0.05).

The changes in SSC of cherry tomatoes stored for 12 days are shown in Figure 10. At day 0, there are no significant differences in SSC between control group and 0.75 wt% CEO group. On day 3, the SSC decreased to 4.16% in control group and 4.96% in 0.75 wt% CEO group, which showed a significant difference (*p* < 0.05). On the 12th day of storage, the difference between the two groups was more significant (*p* < 0.05). The SSC in control group was only 3.52%, while the SSC in 0.75 wt% group could still be maintained at 4.90%. Therefore, the SSC in control group showed an overall downward trend. The SSC of 0.75 wt% CEO group showed a trend of increasing first and then decreasing. The reasons might be as follows. Macromolecular polysaccharide was hydrolyzed into soluble sugar in cherry tomatoes at the initial stage of storage, and the decrease of SSC content at the later stage of storage was related to its participation in respiration as a substrate [40]. However, the SSC value in 0.75 wt% group was significantly more stable, and Lucía Aragüez et al. [41] found that the more stable the SSC value, the better the preservation effect of cherry tomatoes, which is similar to our study.

The results showed that 0.75 wt% CEO Pickering emulsion fresh-keeping paper could substantially inhibit SSC decline in cherry tomatoes, which was attributed to the fact that CEO could inhibit TCA pathway of respiratory metabolism, thus achieving the effect of inhibiting respiratory action [42]. The fresh-keeping paper used in this study could slow down the respiration of cherry tomatoes by decreasing the release of CEO. Meanwhile, the delayed SSC decline of samples using CEO Pickering emulsion paper was associated with its bacteriostatic properties. Nutrient-rich cherry tomatoes are extremely susceptible to infection by various microorganisms such as *Alternaria alternata*. After the infestation of cherry tomatoes, microorganisms proliferated and consumed a large number of nutrients, which resulted in the decline of SSC. CEO has a strong antibacterial effect. The sustained release CEO on the paper made cherry tomatoes achieve a long-term antibacterial effect, and SSC could be kept constant for a long time.

#### 3.2.6. Titratable Acid (TA)

**Figure 11:**
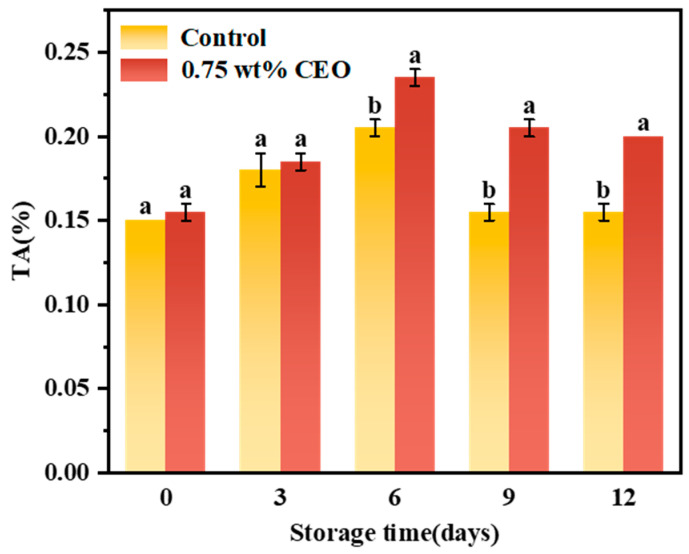
During a 12-day storage period at 25 °C, the effect of CEO Pickering emulsion fresh-keeping paper on titratable acid (TA) of cherry tomatoes. Different superscript letters (a, b) represent statistically significant differences (*p* < 0.05).

As depicted in Figure 11, the TA of control group and 0.75 wt% CEO group both exhibited an increase and followed by a decrease. This was due to the fact that in the early stages of storage, cherry tomato respiration primarily utilized carbohydrates as substrates and produced partial acids through glycolysis and the tricarboxylic acid cycle [43]. At the later stage of storage, cherry tomato respiration began to consume a large amount of acids. On days 0 and 3 of storage, there was no significant difference in TA between control group and 0.75 wt% group. Nonetheless, on day 6 of storage, TA was 0.205% in control group and 0.235% in 0.75 wt% CEO group, showing a significant difference (*p* < 0.05). On day 12, the difference in TA between the two groups was more significant (*p* < 0.05). The TA of control group reduced to 0.155%, while 0.75 wt% CEO group remained at 0.20%. It was proven that 0.75 wt% CEO Pickering emulsion paper had the effect of slowing down the TA decline rate. This might be because the sustained release of CEO on fresh-keeping paper reduced the respiration rate of cherry tomatoes [42]. Meanwhile, the CEO also had an antibacterial effect, which inhibited the consumption of a large amount of acids by microorganisms, so it achieved the purpose of preservation.

#### 3.2.7. Vitamin C (Vc)

**Figure 12:**
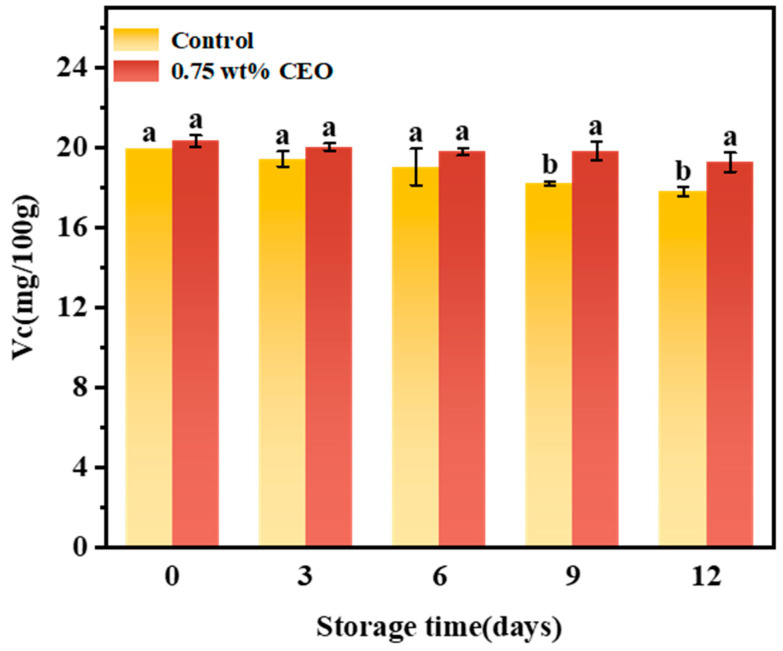
During a 12-day storage period at 25 °C, effect of CEO Pickering emulsion fresh-keeping paper on vitamin C (Vc) of cherry tomatoes. Different superscript letters (a, b) represent statistically significant differences (*p* < 0.05).

Vc, an antioxidant substance, may remove excessive free radicals in body, so it is an important indicator of the preservation effect of fruits and vegetables. Figure 12 shows the changes of Vc in cherry tomatoes stored for 12 days. The Vc content of both control group and 0.75 wt% CEO group showed a decreasing trend because the Vc oxidase of cherry tomatoes might react with oxygen in the air to degrade Vc into dehydroascorbic acid [40]. With storage time, Vc will gradually decrease. In the process of storage, the Vc content of cherry tomatoes in control group and 0.75 wt% CEO groups showed a downward trend, but the decline rate of control group was significantly faster. At days 0, 3 and 6 of storage, there was no significant difference in Vc between control group and 0.75 wt% CEO group. On day 9, Vc decreased to 18.227 mg/100g in control group and 19.830 mg/100 g in 0.75 wt% CEO group, which showed a significant difference (*p* < 0.05). The results showed that the sustained-release CEO on the fresh-keeping paper played a role in protecting antioxidants during storage. CEO also has relatively high antioxidant activity (with DPPH and ABTS scavenging rates of >80% at the concentration of 0.2 g/L) [44]. When free radicals are removed from cherry tomatoes, the loss of antioxidants is reduced which maintains their activity and protects cells from oxidative damage.

#### 3.2.8. Hardness

**Figure 13:**
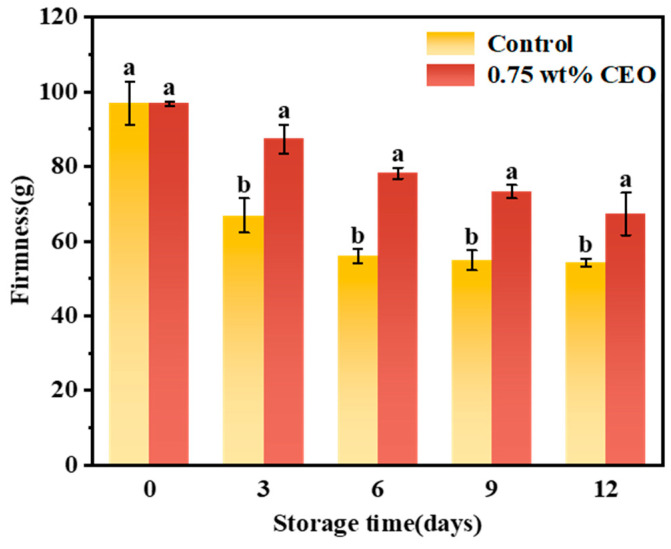
During a 12-day storage period at 25 °C, effect of CEO Pickering emulsion fresh-keeping paper on the hardness of cherry tomato. Different superscript letters (a, b) represent statistically significant differences (*p* < 0.05).

Figure 13 illustrates the hardness changes of cherry tomatoes throughout the storage period. The hardness of both control group and 0.75 wt% CEO group showed a downward trend, which was due to the changes in the cell structure inside the pulp of cherry tomatoes during storage, such as the decomposition of pectin by pectinase in the cells and the rupture of the cell membrane [45]. During storage, there was a significant difference between control group and 0.75 wt% CEO group at day 3 of storage (*p* < 0.05). Among them, the hardness of control group decreased by 31.10%, while the hardness of 0.75 wt% CEO group decreased by only 9.79%, indicating that the fresh-keeping paper had a superior ability to maintain fruit hardness during short-term storage. By the 12th day of storage, hardness decreased by 44.07% in control group and 30.47% in 0.75 wt% CEO group. 

The hardness of control group decreased sharply. On the one hand, because its own respiration and metabolism consumed a large number of nutrients, which resulted in the breakdown of its organizational structure [46]. On the other hand, the microorganisms decomposed a lot of nutrients after invasion due to the lack of autoimmunity in control group. Meanwhile, the microorganisms inside tomatoes may also produce pectinase, which further promotes the decomposition of pectin. Thus, it greatly reduces the hardness of control group. 

The hardness of 0.75 wt% CEO group was maintained. The sustained-release CEO of the fresh-keeping paper can slow down the rate of respiratory action [42]. In addition, CEO has a good antibacterial effect, which can improve the immune ability of cherry tomatoes to microorganisms for a long time. Therefore, the possibility of microbial decomposition of nutrients and the production of pectinase to decompose pectin was reduced. The above results showed that CEO Pickering emulsion fresh-keeping paper had a good ability to maintain fruit hardness, and achieved the goal of preservation.

#### 3.2.9. Microbiological Analysis

**Figure 14:**
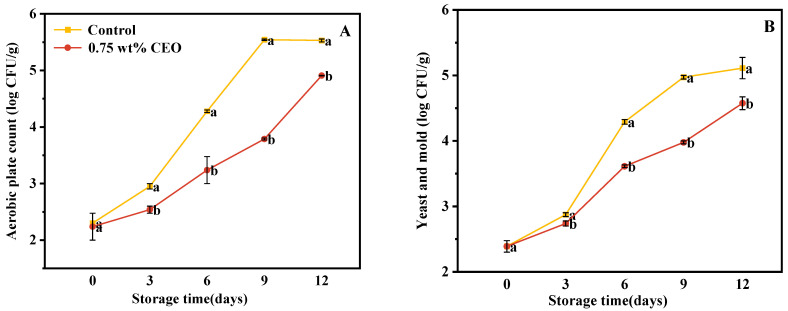
During a 12-day storage period at 25 °C, inhibition of CEO Pickering emulsion fresh-keeping paper on bacteria (**A**), mold and yeast (**B**). Different superscript letters (a, b) represent statistically significant differences (*p* < 0.05).

In this study, the alterations in the population of aerobic bacteria in cherry tomatoes are depicted in Figure 14A. From day 0 to 12 of storage, the number of aerobic bacteria increased in both control group and 0.75 wt% CEO group. At the beginning of storage, the number of aerobic bacteria in control group was 2.301 log CFU/g, 0.75 wt% in the CEO group was (2.24 ± 0.24) log CFU/g, and there was no significant difference between the two groups. On day 3 of storage, there was a significant difference between control group and 0.75 wt% CEO group (*p* < 0.05). The number of aerobic bacteria increased to (2.95 ± 0.05) log CFU/g in control group and (2.54 ± 0.06) log CFU/g in 0.75 wt% CEO group, an increase of 13.39%. On day 9 of storage, control group increased by 140.92%, 0.75 wt% CEO group increased by 69.24%, and the growth rate of control group was nearly twice that of 0.75 wt% CEO group. On day 12, the number of aerobic bacteria increased to (5.53 ± 0.03) log CFU/g in control group and (4.91 ± 0.01) log CFU/g in 0.75 wt% CEO group. Some studies of microbiological analysis on ready-to-use vegetables reported that 6.0 log CFU/g aerobic plate count is preferred to be tolerance limit in vegetables [47]. Therefore, CEO Pickering emulsion fresh-keeping paper had an obvious inhibitory effect on aerobic bacteria. This is due to the significant antibacterial properties of eugenol and the main component in CEO, which exists mainly because of the free hydroxyl group in its structure. Eugenol permeates the cell membrane of bacteria in a non-specific way, inducing damage to the cell structure of *Pseudomonas fluorescens*, *Escherichia coli*, *Staphylococcus aureus*, and other bacteria. The intracellular components are leaked and the hydroxyl group of eugenol can bind to proteins in bacterial cells to promote its inhibitory effect on bacteria [48].

The preservation effect of fresh-keeping paper treatments on cherry tomatoes was investigated by quantifying mold and yeast populations (Figure 14B). From day 0 to 12 of storage, both control group and 0.75 wt% CEO group displayed increasing amounts of mold and yeast, but control group increased at a faster rate. At the beginning of storage, the number of mold and yeast in control group and 0.75 wt% CEO group was (2.389 ± 0.088) log CFU/g, and there was no significant difference between them. On day 3 of storage, there was a significant difference between them (*p* < 0.05). On day 9 of storage, the number of mold and yeast increased to (4.972 ± 0.028) log CFU/g in control group, which increased 108.12%. 0.75 wt% CEO group increased by 66.47%. On day 12, the amount of mold and yeast in control group was (5.112 ± 0.163) log CFU/g, while that in 0.75 wt% CEO group was (4.575 ± 0.098) log CFU/g, with a significant difference (*p* < 0.05). The results indicated that CEO Pickering emulsion paper showed good antifungal activity during storage. This might be because the sustained-release CEO in fresh-keeping paper inhibited the formation of mold and yeast biofilm, and changed their cell morphology. Thus, the growth and reproduction of mold and yeast on cherry tomatoes were inhibited [49].

## 4. Conclusions

In this study, a novel functional fresh-keeping paper was developed through Pickering emulsion. Our study proved that 0.75 wt% CEO Pickering emulsion had a certain stability, and the prepared fresh-keeping paper has good performance of antioxidant activity and sustained-release CEO. Moreover, cherry tomatoes were treated with 0.75 wt% CEO Pickering emulsion fresh-keeping paper, and the characteristics of cherry tomatoes during storage were investigated. It effectively preserved the postharvest quality of the cherry tomatoes. Therefore, 0.75 wt% CEO Pickering emulsion fresh-keeping paper not only has stable characteristics, but also achieved the purpose of preserving cherry tomato freshness. Our research is promising and provides a novel fresh-keeping material for fruit and vegetable preservation in the future.

## Figures and Tables

**Table 1:** Samples: 0 wt% composite colloidal particle dispersion and 0.75 wt% was Pickering emulsion with CEO. Different superscript letters indicate significant differences (*p* < 0.05).

Sample	Interfacial Tension (mNm^−1^)	CI (%)	ζ-Potential (mv)	Antioxidant Assay (%)	Particle Size (d.nm)
0 wt%	33.194 ± 0.338 ^a^	96.88 ± 0.462 ^b^	36.6 ± 1.768 ^a^	55.2 ± 0.019 ^b^	399.63 ± 3.571 ^b^
0.75 wt%	32.360 ± 0.329 ^b^	98.85 ± 0.165 ^a^	11.7667 ± 0.834 ^b^	87.7 ± 0.042 ^a^	463.47 ± 4.177 ^a^

## Data Availability

The original contributions presented in the study are included in the article, further inquiries can be directed to the corresponding author.
